# Mapping research themes and collaborative networks in schizophrenia genetics and clinical psychological correlates: A bibliometric and scientometric study (1957–2025)

**DOI:** 10.1097/MD.0000000000049245

**Published:** 2026-06-12

**Authors:** Mahdi Naeim, Mohammad Narimani, Seifollah Aghajani

**Affiliations:** a Department of Psychology, Faculty of Educational Sciences and Psychology, University of Mohaghegh Ardabili, Ardabil, Iran.

**Keywords:** bibliometrics, genetics, research trends, schizophrenia, scientometrics, VOSviewer

## Abstract

**Background:**

This study presents a bibliometric and scientometric analysis of research trends in schizophrenia genetics over nearly 7 decades (1957–2025). The field has evolved from early heritability studies to genome-wide association studies and multi-omics approaches. The objective was to map the historical trajectory, thematic evolution, key contributors, and global collaboration patterns in relation to clinically relevant psychological constructs.

**Methods:**

An integrated bibliometric and scientometric approach was applied. A total of 5679 publications were included after systematic retrieval and PRISMA-guided screening from Scopus, PubMed, and Web of Science (initial n = 6193; final n = 5679). Data preprocessing included deduplication, keyword normalization, and metadata verification. VOSviewer (version 1.6.20) was used to construct co-authorship, keyword co-occurrence, and source co-citation networks. Thresholds were defined empirically to balance network interpretability and coverage.

**Results:**

The findings suggest a gradual increase in research activity beginning in the 1990s, followed by a marked acceleration after 2010. Four thematic clusters were identified: neurobiological mechanisms and endophenotypes; basic genetic foundations; pharmacological treatments; and clinical comorbidities. Temporal patterns indicate a shift between 2012 and 2016 from genetic association studies toward functional genomics and cognitive neuroscience. Prominent contributors include J. van Os, R.E. Gur, and M.T. Tsuang. The United States may play a prominent role within the global collaboration network. However, clinically relevant psychological constructs may be less centrally integrated within the network structure.

**Conclusion:**

This study provides a structured bibliometric mapping of schizophrenia genetics research from 1957 to 2025. The findings may suggest increasing movement toward large-scale, consortium-based research models and a possible partial separation between clinical and basic science domains. Importantly, the results highlight a relative underrepresentation of clinically interpretable psychological dimensions. These findings may indicate the need for stronger integration between genetic discoveries and clinical psychological frameworks to enhance translational relevance.

## 1. Introduction

Schizophrenia is a severe and heterogeneous psychiatric disorder that affects approximately 0.3 to 1% of the global population and is considered a major public health challenge worldwide.^[1–3]^ The disorder is characterized by positive symptoms (e.g., hallucinations and delusions), negative symptoms, cognitive deficits, and functional impairments. These features are associated with substantial disruption in psychosocial functioning and quality of life.^[1]^ In 2019, schizophrenia accounted for an estimated 23.6 million cases globally and contributed to 12.66 million disability-adjusted life years, indicating a considerable global burden.^[4,5]^ Beyond its chronic course, schizophrenia is associated with significant direct and indirect costs, including long-term healthcare utilization and productivity loss.^[6]^ These observations highlight the need for integrative research approaches that link biological mechanisms with clinically observable psychological phenomena.^[7]^

Over the past 2 decades, genetic research has contributed substantially to the understanding of schizophrenia etiology. Large-scale genome-wide association studies (GWAS) suggest that schizophrenia is a highly polygenic disorder. Risk appears to arise from the cumulative effects of numerous common variants, alongside rare copy number variants and rare deleterious mutations.^[8,9]^ Seminal work by Owen and O’Donovan (2023) suggests that most heritability is attributable to common alleles, while rare variants may exert stronger but less frequent effects.^[8]^ Genes such as NRG1 and DISC1, as well as shared genetic architectures with bipolar disorder and other neurodevelopmental conditions, have been reported.^[10,11]^ More recent exome sequencing studies have identified rare variants in genes such as SETD1A as potential contributors to schizophrenia risk.^[9]^ Despite these advances, the translation of genetic findings into clinically meaningful psychological frameworks remains limited. Genetic results are increasingly examined in relation to symptom dimensions, cognitive impairments, and psychopathological profiles, but this integration remains partial and fragmented.^[12]^

Bibliometric and scientometric analyses offer a systematic and quantitative framework for examining the evolution of scientific knowledge. These approaches can be used to identify influential studies, map research themes, and visualize collaboration networks across disciplines and regions.^[13–16]^ Visualization tools such as VOSviewer are widely used to analyze co-authorship patterns, keyword co-occurrence, and citation structures in complex research domains.^[17–20]^ In schizophrenia research, Jin et al (2025) used VOSviewer to map biomarker-related studies and identified thematic clusters centered on genetics, proteomics, and genes such as NRXN1 and CNTNAP2.^[13]^ Similarly, a recent bibliometric study (2024) examined global research on schizophrenia and serotonin and reported the prominence of pharmacogenetics and epigenetics.^[17]^ However, these studies primarily focus on molecular and biological dimensions and provide limited analysis of clinical psychological relevance.

Despite these advances, a critical gap remains in the literature. The integration of genetic discoveries with clinically meaningful psychological constructs appears insufficient. Existing bibliometric studies have largely emphasized molecular and biological aspects of schizophrenia. Less attention has been given to their relationship with symptomatology, cognitive dysfunction, and broader psychopathological profiles. This imbalance may limit the interpretability and translational value of genetic research in clinical contexts. Given the complexity of schizophrenia as a disorder at the intersection of genetics, brain function, and clinical psychopathology, there is a need for analytically focused mapping approaches that prioritize clinically relevant psychological dimensions. Bibliometric analysis can help identify research hotspots, emerging trends, and underexplored areas. It can also reveal patterns of international and institutional collaboration that shape knowledge production in this field.^[17]^

Accordingly, the present study aims to provide a bibliometric and scientometric analysis of schizophrenia genetics with a specific focus on its integration with clinical psychological correlates. Rather than offering a purely descriptive overview, this study emphasizes the alignment between genetic findings and clinically interpretable psychological constructs. By mapping research themes, collaboration networks among authors, institutions, and countries, and temporal trends from 1957 to 2025, this study seeks to clarify how genetic research relates to clinically relevant psychological domains. The findings are intended to identify dominant and emerging research trajectories, highlight gaps in clinical integration, and inform future interdisciplinary efforts aimed at improving the translational relevance of psychiatric genetics.

## 2. Materials and methods

### 2.1. Data sources

This bibliometric and scientometric study was conducted using publication records retrieved from 3 major databases: PubMed, Scopus, and Web of Science (WoS). These databases were selected due to their complementary coverage of biomedical, psychiatric, and psychological literature and their established use in bibliometric research.

The study period covered publications from January 1, 1957 to December 31, 2025. The search was executed on January 12, 2026. This period was selected to capture the full developmental trajectory of schizophrenia genetics research, from early heritability and family-based studies to modern genomic approaches, including GWAS, polygenic risk scores (PRS), and multi-omics integration.

All retrieved records were exported from each database and merged into a unified dataset prior to analysis. Duplicate records across databases were identified and removed. No manual content modification or enrichment of bibliographic records was performed. The final dataset was used for all subsequent analyses.

Ethical approval and informed consent were not required because the study was based exclusively on publicly available bibliographic data and did not involve human participants.

### 2.2. Search strategy

A systematic and reproducible search strategy was implemented across Scopus, PubMed, and Web of Science to identify literature at the intersection of schizophrenia genetics and clinically relevant psychological constructs. The search strategy was designed to ensure simultaneous inclusion of genetic/genomic content and clinical psychological relevance.

The search framework included 4 domains of genetic research: core genetic concepts, sequencing technologies, genetic architectures, and functional genomics. These were operationalized using the following terms:

“genetic*” OR “genomic*” OR “gene*” OR “allele*” OR “heritability” OR “mutation*” OR “GWAS” OR “genome-wide association” OR “polygenic risk score*” OR “PRS” OR “epigenetic*” OR “epigenomic*” OR “pharmacogenetic*” OR “pharmacogenomic*” OR “next generation sequencing” OR “NGS” OR “whole exome sequencing” OR “WES” OR “whole genome sequencing” OR “WGS” OR “copy number variation” OR “CNV.”

These genetic terms were combined with clinically oriented psychological terms, including “clinical psychology” OR “psychopathology” OR “clinical phenotype” OR “symptom*.”

The full search syntax was adapted for each database as follows:

#### 2.2.1. Scopus

TITLE-ABS-KEY (“schizophrenia”) AND TITLE-ABS-KEY (genetic* OR genomic* OR gene* OR allele* OR heritability OR mutation* OR GWAS OR “genome-wide association” OR “polygenic risk score*” OR PRS OR epigenetic* OR epigenomic* OR pharmacogenetic* OR pharmacogenomic* OR “next generation sequencing” OR NGS OR “whole exome sequencing” OR WES OR “whole genome sequencing” OR WGS OR “copy number variation” OR CNV) AND TITLE-ABS-KEY (“clinical psychology” OR “psychopathology” OR “clinical phenotype” OR “symptom*”).

#### 2.2.2. PubMed

(“schizophrenia”[Title/Abstract]) AND (genetic*[Title/Abstract] OR genomic*[Title/Abstract] OR gene*[Title/Abstract] OR allele*[Title/Abstract] OR heritability[Title/Abstract] OR mutation*[Title/Abstract] OR GWAS[Title/Abstract] OR “genome-wide association”[Title/Abstract] OR “polygenic risk score*”[Title/Abstract] OR PRS[Title/Abstract] OR epigenetic*[Title/Abstract] OR epigenomic*[Title/Abstract] OR pharmacogenetic*[Title/Abstract] OR pharmacogenomic*[Title/Abstract] OR “next generation sequencing”[Title/Abstract] OR NGS[Title/Abstract] OR “whole exome sequencing”[Title/Abstract] OR WES[Title/Abstract] OR “whole genome sequencing”[Title/Abstract] OR WGS[Title/Abstract] OR “copy number variation”[Title/Abstract] OR CNV[Title/Abstract]) AND (“clinical psychology”[Title/Abstract] OR “psychopathology”[Title/Abstract] OR “clinical phenotype”[Title/Abstract] OR “symptom*”[Title/Abstract]).

#### 2.2.3. Web of Science (WoS)

TS=(“schizophrenia”) AND TS = (genetic* OR genomic* OR gene* OR allele* OR heritability OR mutation* OR GWAS OR “genome-wide association” OR “polygenic risk score*” OR PRS OR epigenetic* OR epigenomic* OR pharmacogenetic* OR pharmacogenomic* OR “next generation sequencing” OR NGS OR “whole exome sequencing” OR WES OR “whole genome sequencing” OR WGS OR “copy number variation” OR CNV) AND TS=(“clinical psychology” OR “psychopathology” OR “clinical phenotype” OR “symptom*”).

Across all databases, the search was limited to English-language publications classified as Articles or Reviews and published between 1957 and 2025. Non-peer-reviewed sources, including editorials, conference abstracts, book chapters, and errata, were excluded.

### 2.3. Inclusion and exclusion criteria

A two-stage screening process was applied, consisting of title/abstract screening followed by full-text screening.

Studies were included if they met all of the following criteria: they addressed schizophrenia with explicit integration of genetic/genomic content and clinical psychological features such as symptoms, psychopathology, or cognitive outcomes; they were classified as Articles or Reviews; and they were published in English between 1957 and 2025.

Studies were excluded if they were limited to non-human, molecular, or cellular models without clinical psychological relevance; if they were non-peer-reviewed publication types such as editorials, letters, conference abstracts, book chapters, or case reports; or if they did not include a substantive genetic or genomic component.

### 2.4. Data processing and cleaning

All records retrieved from PubMed, Scopus, and Web of Science were merged into a single dataset. Duplicate records were removed using a hierarchical procedure based on DOI matching, followed by title and author similarity checks for records without DOI.

**Figure 1.**
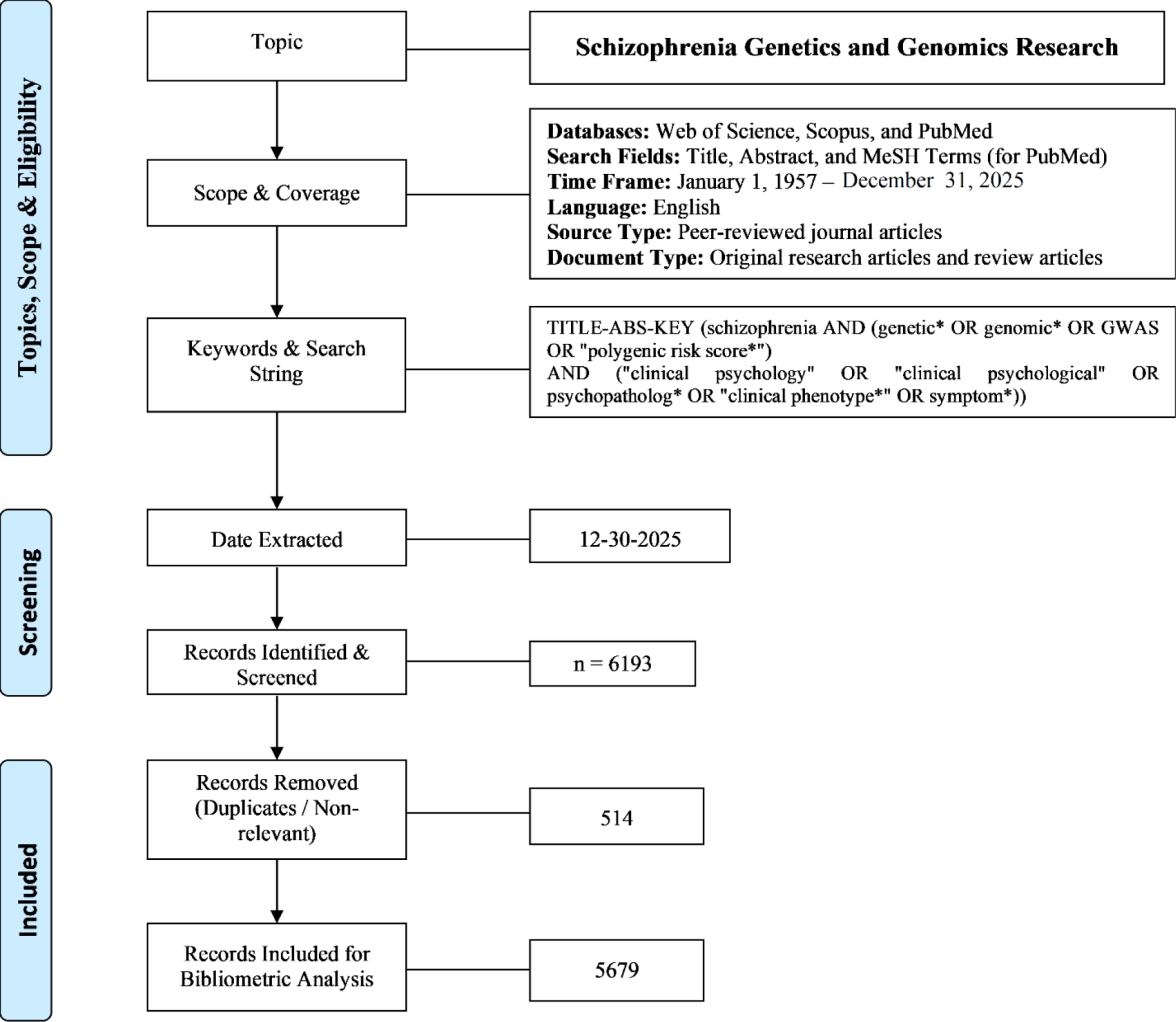
Identification of studies via databases and registers (PRISMA flow diagram).

After deduplication, records were screened according to predefined eligibility criteria. The final dataset consisted of eligible publications included in the bibliometric analysis (Fig. 1).

Bibliographic data were exported in CSV and RIS formats. Data preprocessing was performed using Microsoft Excel and Python (pandas library). This included standardization of author names, harmonization of keywords, and validation of metadata completeness.

Keyword normalization was applied to reduce redundancy. This included merging of synonyms (e.g., “GWAS” and “genome-wide association study”), standardization of singular and plural forms, and removal of non-informative terms. Spelling variants were harmonized to ensure consistency in co-occurrence analysis.

A minimum keyword occurrence threshold of 5 was applied for inclusion in co-occurrence analysis.

### 2.5. Network mapping procedure

#### 2.5.1. Bibliometric analysis and visualization

Bibliometric analyses were conducted using VOSviewer (version 1.6.20). The software was used to construct and visualize co-authorship, co-occurrence, citation, and co-citation networks.

Co-authorship analysis was used to examine collaboration patterns among authors, institutions, and countries. Keyword co-occurrence analysis was used to identify thematic structures. Citation and co-citation analyses were used to identify influential publications and intellectual structures within the field.

Minimum thresholds were applied to improve interpretability and reduce noise. The following thresholds were used:

- Minimum documents per author: 3
- Minimum citations per author: 10
- Minimum keyword occurrences: 5
- Minimum documents per country: 5

All analyses were conducted on the unified dataset derived from the 3 databases.

#### 2.5.2. Network construction and clustering

Network construction followed a standardized workflow, including data extraction, preprocessing, and import into VOSviewer.

Clustering was performed using the association strength normalization method. The resolution parameter was set to 1.00 for all analyses to ensure comparability across networks. Default VOSviewer layout parameters were used (attraction = 2, repulsion = −1).

Cluster stability was assessed through repeated runs with identical parameters. Only stable cluster configurations were retained for interpretation.

Cluster interpretation was conducted in a structured analytic manner to identify thematic relationships and research gaps rather than descriptive labeling.

All preprocessing steps, thresholds, and analytical parameters were applied consistently across all analyses to ensure transparency, reproducibility, and methodological rigor.

## 3. Results

### 3.1. Overview of publications

A total of 6193 records were initially identified across 3 major databases, including Scopus (n = 2845), PubMed (n = 1876), and Web of Science (n = 1472), through a comprehensive search conducted on January 12, 2026. All retrieved records were exported and merged into a unified dataset for further processing.

Following integration, duplicates were removed using DOI matching and bibliographic comparison. The remaining records were screened by title and abstract, followed by full-text assessment based on predefined inclusion and exclusion criteria. In total, 514 records were excluded, resulting in a final dataset of 5679 publications.

The detailed study selection process is presented in Figure 1 (PRISMA flow diagram), ensuring transparency and reproducibility.

**Figure 2.**
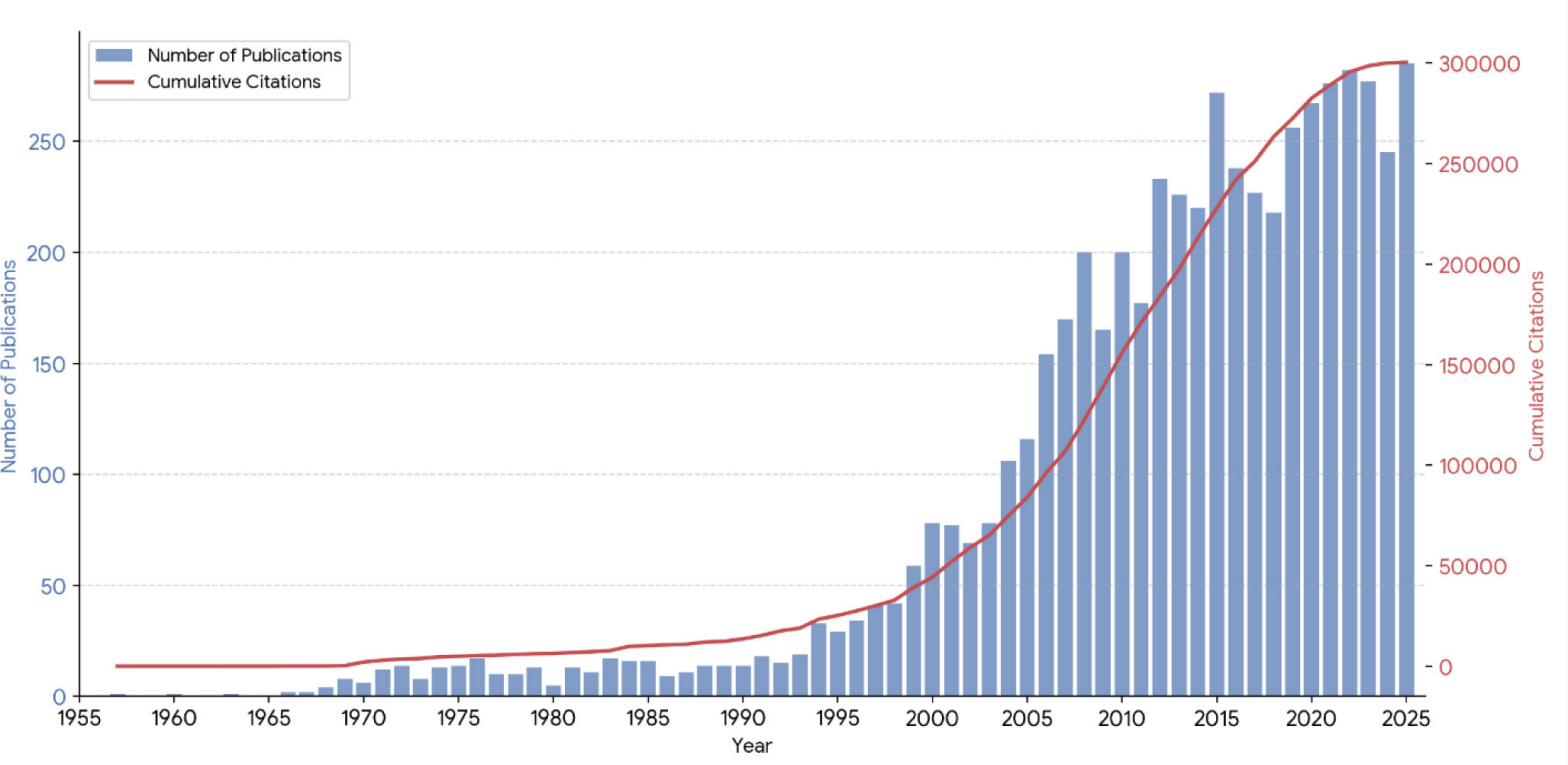
Annual publication trend and citation growth (1957–2025).

According to Figure 2, publication and citation trends from 1957 to 2025 show a non-linear developmental pattern. Early activity (1957–1980s) was limited and mainly based on heritability and family studies. From the 1990s onward, a steady increase is observed, corresponding to the introduction of molecular genetics. A marked acceleration occurs after 2010, aligned with the rise of GWAS and large-scale collaborative projects.

Overall, the trajectory suggests a shift toward large-scale, data-driven, and collaborative research structures rather than simple linear growth. Citation accumulation follows a cumulative pattern consistent with the delayed impact of influential studies.

### 3.2. Leading authors

Table 1 identifies the most prolific contributors in schizophrenia genetics research from 1957 to 2025. The output is highly concentrated among a small number of authors, with J. van Os (78 publications), M. T. Tsuang (58), M. J. Owen (54), and R. E. Gur (51) as leading contributors.

**Table 1:** Highlights the top contributing authors in the field.

Author	Publications
van Os, J.	78
Tsuang, M. T.	58
Serretti, A.	53
Gur, R. E.	51
Owen, M. J.	54
de Haan, L.	44
Seidman, L. J.	42
O’donovan, M. C.	38
Gur, R. C.	34
Calkins, M. E.	31

This distribution indicates a strongly skewed productivity pattern consistent with bibliometric concentration effects such as Lotka law. It suggests that a limited group of senior researchers has played a central role in shaping the intellectual and collaborative development of the field across different methodological eras.

### 3.3. International collaboration and institutional contributions

Institutional analysis shows that schizophrenia genetics research is mainly concentrated in major academic and clinical centers in North America and Europe. Key institutions include King’s College London, Centro de Investigación Biomédica en Red de Salud Mental, University of Toronto, Harvard Medical School, and Icahn School of Medicine at Mount Sinai.

Rather than being only descriptive, this concentration likely reflects structural advantages such as access to large cohorts, genomic infrastructure, and participation in international consortia, which collectively facilitate high-impact research production.

### 3.4. Network analysis using VOSviewer

**Figure 3.**
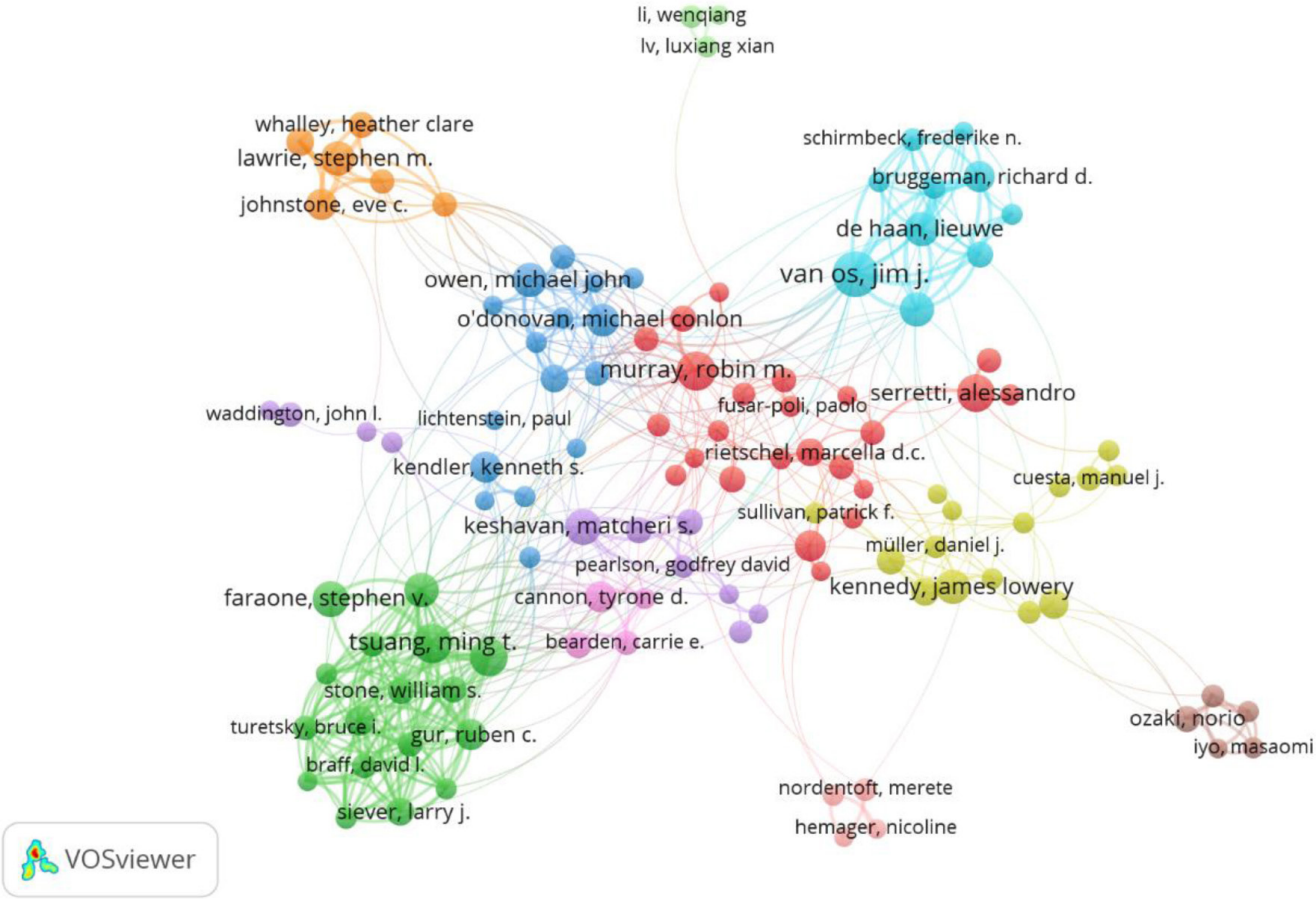
Co-authorship network of authors.

As shown in Figure 3, the co-authorship network reveals a highly collaborative but unevenly structured research system. Collaboration patterns largely reflect geographical and institutional proximity. Highly connected authors, including van Os and Murray, act as central nodes within the network, indicating the importance of key hubs in maintaining large collaborative structures. Overall, the structure suggests increasing dependence on international and consortium-based research designs.

**Figure 4.**
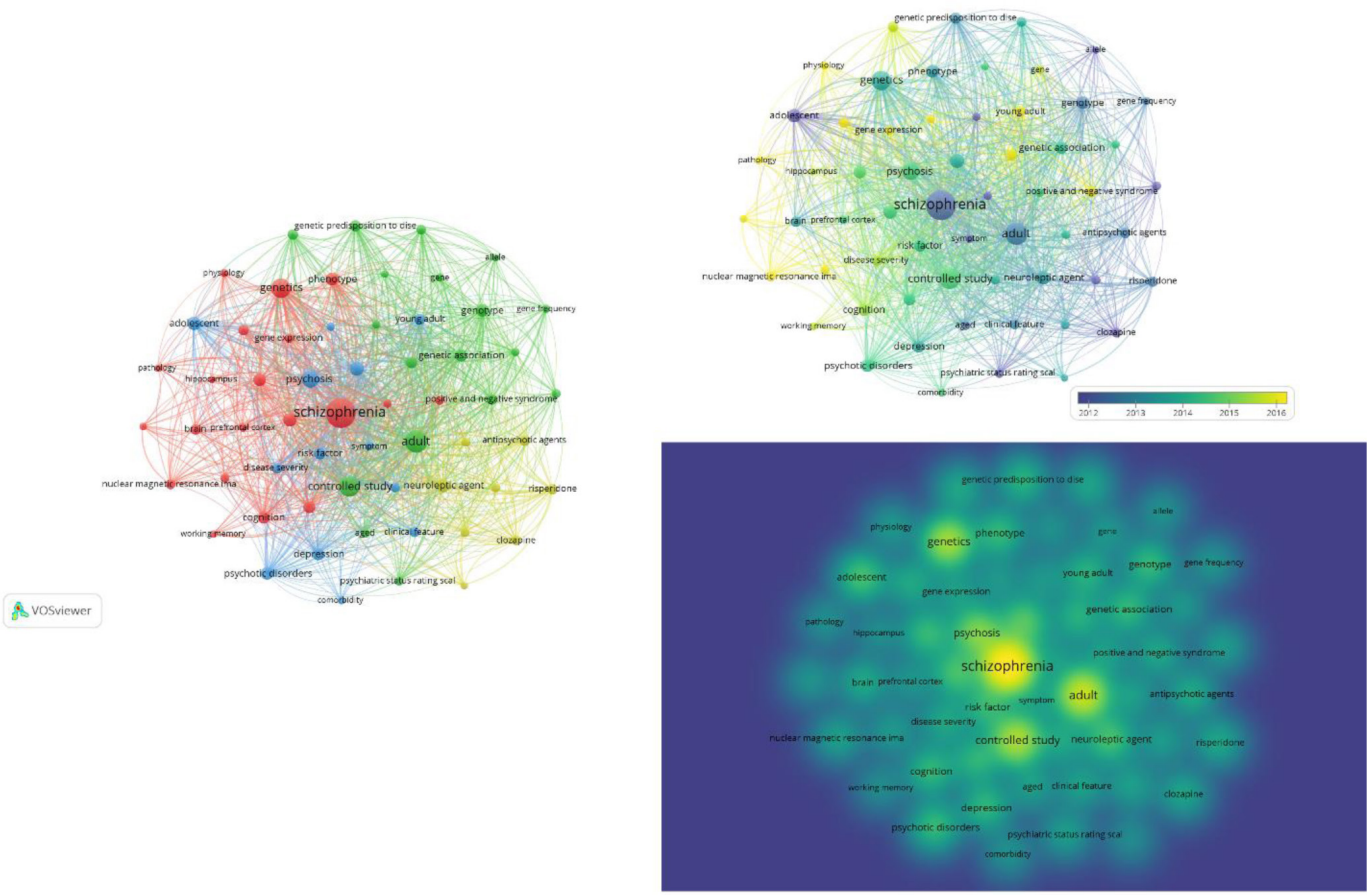
Co-occurrence network, temporal evolution, and keyword density map.

Figure 4 presents the thematic structure of the field. Schizophrenia functions as the central linking concept across 4 main domains: neurobiological mechanisms, genetic foundations, pharmacological research, and clinical manifestations.

A key observation is that genetic and neurobiological domains are more densely interconnected, while clinically relevant psychological constructs (e.g., symptoms, cognition) are less central within the network. Pharmacological research occupies an intermediate position between these domains.

Temporal patterns suggest a shift (approximately 2012–2016) from association-based genetic studies toward functional genomics and cognitive neuroscience, indicating a transition toward a mechanistic interpretation of genetic variation. Overall, the structure suggests partial integration between genetic and clinical psychological domains, but with a remaining imbalance in connectivity.

**Figure 5.**
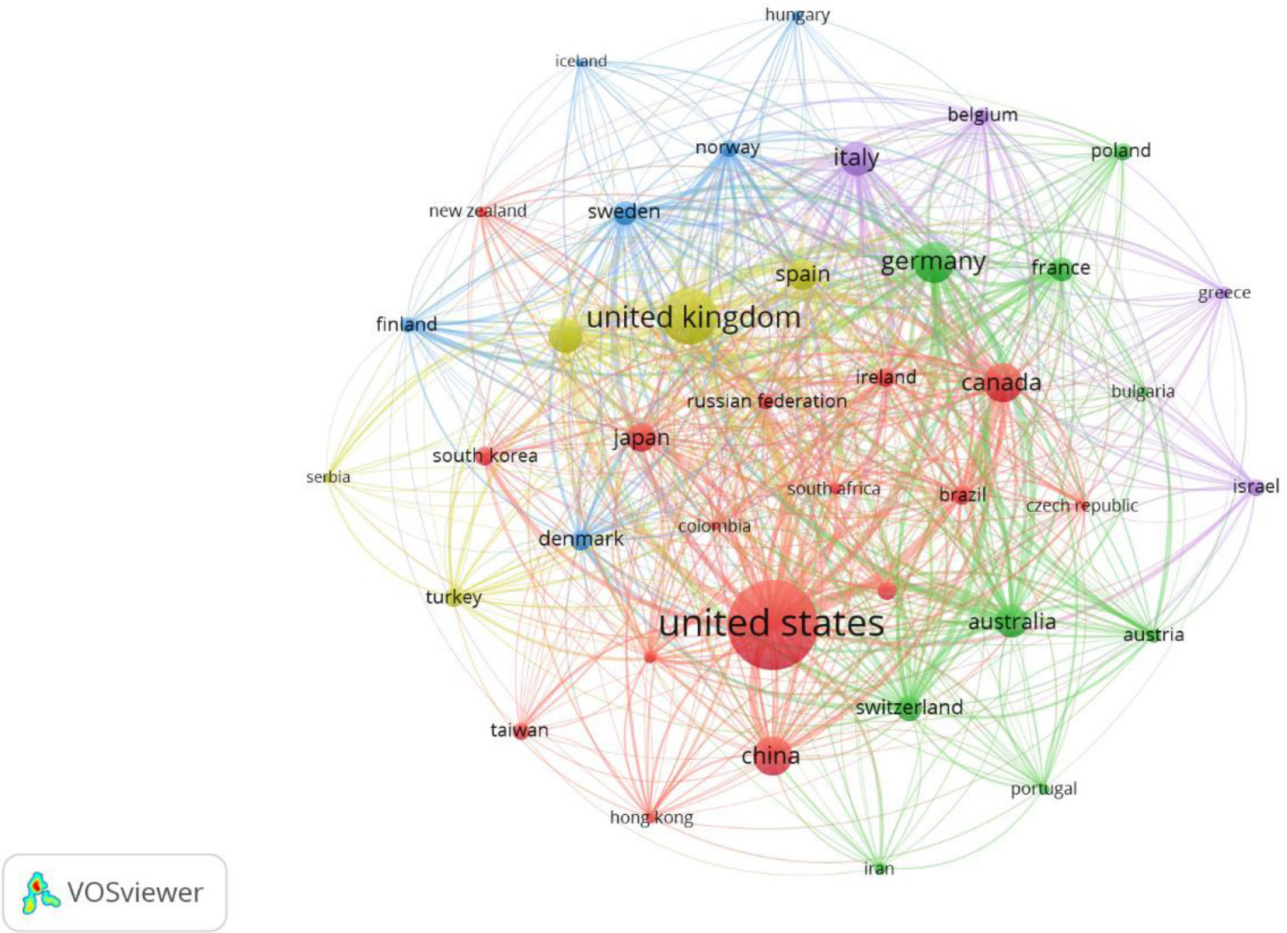
International collaboration network.

As shown in Figure 5, the international collaboration network is globally connected but structurally uneven. The United States occupies a central position in terms of productivity and connectivity, while Europe and Asia form secondary regional clusters. The United Kingdom appears to function as a bridging node between major collaboration regions. This pattern suggests a core–periphery structure influenced by differences in research infrastructure and funding capacity.

**Figure 6.**
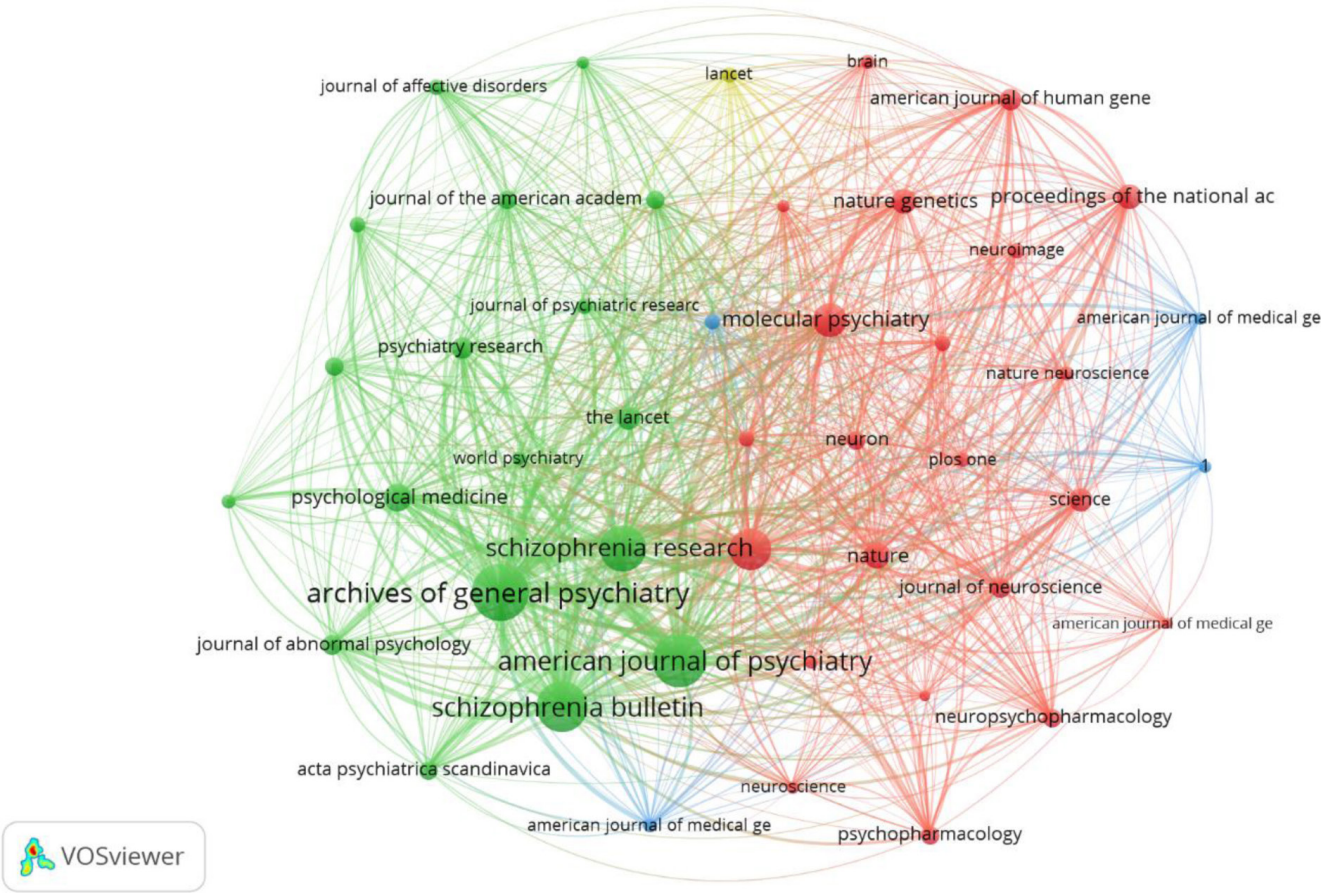
Co-citation network of scientific sources.

Figure 6 shows the co-citation network and reveals 2 partially distinct intellectual domains. One is centered on clinical psychiatry journals such as *JAMA Psychiatry*, the *American Journal of Psychiatry*, and *Schizophrenia Bulletin*. The second is focused on molecular genetics and neuroscience journals such as *Nature Genetics*, *Molecular Psychiatry*, and *Neuron*.

*Schizophrenia Research* occupies an intermediate position, suggesting a partial translational link between clinical and basic science literature. Overall, the structure indicates the parallel development of clinical and molecular research streams with limited but emerging integration.

## 4. Discussion

This bibliometric analysis examines trends in schizophrenia genetics research over a long period (1957–2025). The results indicate a gradual and non-linear increase in scientific output. Early publications were limited and mainly based on family and heritability studies. From around 2010 onward, publication output increased more rapidly. This change may coincide with the widespread use of high-throughput genomic methods, including GWAS, next-generation sequencing (NGS), and multi-omics approaches.^[21,22]^ Overall, this pattern may reflect a shift toward data-intensive, consortium-based, and computationally driven research rather than simple numerical expansion.

The co-authorship structure (Table 1 and Fig. 3) suggests a concentrated pattern of scientific productivity. A relatively small group of researchers, including van Os, Gur, Tsuang, and Owen, appears to show sustained contributions over time. This distribution may be consistent with skewed productivity patterns described by Lotka law. The network structure may reflect increasing reliance on collaborative and consortium-based research models. The observed geographical clustering (e.g., Europe vs North America) may be shaped by institutional proximity, funding structures, and historical collaboration pathways rather than strict thematic separation. This pattern may be consistent with the increasing need for large sample sizes, cross-cohort replication, and statistical robustness in psychiatric genetics research.^[23]^

The keyword co-occurrence analysis (Fig. 4) identifies 4 main thematic clusters: neurobiological mechanisms, genetic foundations, pharmacological treatment, and clinical comorbidities. These clusters show partial overlap, suggesting limited but increasing conceptual interaction across domains. The temporal overlay may indicate a shift around 2012 to 2016 from association-based genetic studies toward functional genomics and cognitive neuroscience. This transition may reflect a movement from gene discovery toward pathway-level and mechanism-oriented interpretation of genetic variation.^[24,25]^ Despite this evolution, the integration of clinical psychological constructs remains incomplete. Symptoms, cognition, and psychopathological dimensions are present in the literature but remain less central within the overall genetic research structure. This suggests that translation from genetic findings to clinically operational psychological frameworks is still developing and uneven across subdomains.

A key contribution of this study is the explicit mapping of the structural gap between genetic research and clinically relevant psychological constructs. Unlike previous bibliometric analyses that primarily emphasize molecular outputs, this study highlights the relative peripheral positioning of clinical psychological variables within the research network. This finding does not indicate the absence of clinical relevance, but rather may reflect partial and heterogeneous integration across domains. It may help clarify why translation from genetic discovery to clinical application remains limited in schizophrenia research.

The citation and co-citation networks (Fig. 6) reveal a structured intellectual separation between clinical psychiatry journals (e.g., JAMA Psychiatry) and basic science outlets (e.g., Nature Genetics). Schizophrenia Research appears to occupy an intermediate position, potentially functioning as a partial translational bridge between these domains. This configuration may suggest a partially compartmentalized knowledge structure, in which clinical and molecular research streams evolve in parallel with only partial integration. However, the presence of bridging journals may indicate that cross-domain knowledge exchange does occur, although it is not fully systematic.^[26,27]^

International collaboration patterns (Fig. 5) indicate a globally connected but uneven research structure. The United States occupies a central position in terms of productivity and network connectivity. European and Asian countries form regional clusters with varying degrees of integration into global networks. This pattern may reflect differences in funding capacity, research infrastructure, and access to large-scale datasets.^[28,29]^ The observed structure may be interpreted as a dynamic core–periphery system rather than a fixed hierarchy, reflecting evolving international research dependencies.

From a clinical psychological perspective, symptom dimensions, cognitive impairment, and treatment-related variables are present within the literature but do not function as central organizing constructs. Instead, they appear in more specialized or secondary positions within thematic networks. This pattern suggests that the integration of genetic architecture with clinically meaningful psychological frameworks remains incomplete. Consequently, the translational pathway from genomic findings to clinical psychological models is still in an intermediate developmental stage.

Several limitations should be acknowledged. The exclusion of non-English publications and gray literature may introduce selection bias. Bibliometric methods rely on metadata and therefore cannot capture the full conceptual depth of individual studies. Network mapping approaches, including VOSviewer, are sensitive to preprocessing decisions, synonym handling, and parameter selection.^[18,30]^ Citation-based metrics are also influenced by time-lag effects, particularly for recent publications. Differences across databases may affect the coverage and harmonization of records. Accordingly, findings should be interpreted as structured descriptive patterns rather than definitive representations of the field.

Future research may benefit from integrating bibliometric approaches with systematic or qualitative methods to improve interpretative depth. Combining network-based mapping with content-level analysis may clarify how genetic findings relate to clinical phenotypes. More focused investigations on translational domains such as cognitive genomics or functional pathways underlying symptom dimensions may help identify clinically actionable mechanisms. Inclusion of preprint databases and regional indexing systems may also improve coverage. Finally, bibliometric findings should be interpreted as exploratory and descriptive; the observed patterns reflect tendencies in the literature rather than causal relationships.

## 5. Conclusion

This bibliometric analysis offers a comprehensive overview of global research trends in schizophrenia genetics from 1957 to 2025. The findings suggest a marked increase in publication activity, particularly after 2010, reflecting the impact of the genomic era. Influential authors, including van Os, Gur, and Tsuang, emerged as central contributors, while the United States, followed by the United Kingdom and Germany, demonstrated prominent roles within a stratified global collaboration network. Thematic clustering identified a clear evolution from basic association studies to more complex investigations of neurobiology and cognition. The citation networks highlighted both the separation and interaction between clinical and basic science domains, emphasizing the importance of translational interfaces. Overall, the findings may indicate an ongoing transition toward more integrative and mechanism-oriented research approaches, although gaps between genetic discovery and clinical application remain. In particular, the integration of genetic findings with symptom-level and cognitive psychological constructs appears to be underdeveloped, highlighting an important direction for future interdisciplinary research. This study suggests the value of bibliometric mapping tools such as VOSviewer in visualizing complex research structures and guiding strategic scientific inquiry. Future research should prioritize approaches that explicitly link genetic variation to symptom-level and cognitive outcomes, thereby enhancing the clinical relevance and translational impact of psychiatric genomics. Future work should focus on enhancing cross-domain integration and addressing structural inequalities in global research participation, thereby supporting more inclusive and clinically relevant advances in psychiatric genetics.

## Author contributions

**Conceptualization:** Mahdi Naeim, Mohammad Narimani, Seifollah Aghajani.

**Data curation:** Mahdi Naeim, Mohammad Narimani, Seifollah Aghajani.

**Formal analysis:** Mahdi Naeim, Mohammad Narimani, Seifollah Aghajani.

**Funding acquisition:** Mahdi Naeim, Mohammad Narimani, Seifollah Aghajani.

**Investigation:** Mahdi Naeim, Mohammad Narimani, Seifollah Aghajani.

**Methodology:** Mahdi Naeim, Mohammad Narimani, Seifollah Aghajani.

**Project administration:** Mahdi Naeim, Mohammad Narimani, Seifollah Aghajani.

**Resources:** Mahdi Naeim, Mohammad Narimani, Seifollah Aghajani.

**Software:** Mahdi Naeim, Mohammad Narimani, Seifollah Aghajani.

**Supervision:** Mahdi Naeim, Mohammad Narimani, Seifollah Aghajani.

**Validation:** Mahdi Naeim, Mohammad Narimani, Seifollah Aghajani.

**Visualization:** Mahdi Naeim, Mohammad Narimani, Seifollah Aghajani.

**Writing – original draft:** Mahdi Naeim, Mohammad Narimani, Seifollah Aghajani.

**Writing – review & editing:** Mahdi Naeim, Mohammad Narimani, Seifollah Aghajani.
